# Pathogenesis of *Y. enterocolitica* and *Y. pseudotuberculosis* in Human Yersiniosis

**DOI:** 10.4061/2011/182051

**Published:** 2011-09-12

**Authors:** Cristi L. Galindo, Jason A. Rosenzweig, Michelle L. Kirtley, Ashok K. Chopra

**Affiliations:** ^1^Department of Microbiology & Immunology, Sealy Center for Vaccine Development, Institute of Human Infections & Immunity, and the Galveston National Laboratory, University of Texas Medical Branch, 301 University Boulevard, Galveston, TX 77555-1070, USA; ^2^Department of Biology, Center for Bionanotechnology and Environmental Research (CBER), Texas Southern University, 3100 Cleburne Street, Houston, TX 77004, USA

## Abstract

Yersiniosis is a food-borne illness that has become more prevalent in recent years due to human transmission via the fecal-oral route and prevalence in farm animals. Yersiniosis is primarily caused by *Yersinia enterocolitica* and less frequently by *Yersinia pseudotuberculosis*. Infection is usually characterized by a self-limiting acute infection beginning in the intestine and spreading to the mesenteric lymph nodes. However, more serious infections and chronic conditions can also occur, particularly in immunocompromised individuals. *Y. enterocolitica* and *Y. pseudotuberculosis* are both heterogeneous organisms that vary considerably in their degrees of pathogenicity, although some generalizations can be ascribed to pathogenic variants. Adhesion molecules and a type III secretion system are critical for the establishment and progression of infection. Additionally, host innate and adaptive immune responses are both required for yersiniae clearance. Despite the ubiquity of enteric *Yersinia* species and their association as important causes of food poisoning world-wide, few national enteric pathogen surveillance programs include the yersiniae as notifiable pathogens. Moreover, no standard exists whereby identification and reporting systems can be effectively compared and global trends developed. This review discusses yersinial virulence factors, mechanisms of infection, and host responses in addition to the current state of surveillance, detection, and prevention of yersiniosis.

## 1. Introduction

Yersiniosis is typically a self-limiting, gastrointestinal disease of global concern. However, despite the known association of the causative agents (*Y. enterocolitica*, YE, and very rarely *Y. pseudotuberculosis*, YPT) with both gastroenteritis and extraintestinal infections, it remains a poorly understood disease. Sporadic cases are still reported in which food is not suspected as the source of infection, and isolation from contaminated food sources is often problematic. Because yersiniosis is considered relatively uncommon and YE and YPT are ubiquitous, food and water supplies are not regularly monitored for these bacterial pathogens. However, the ability of the yersiniae to persist in a nonculturable but viable state in natural samples [1] and to grow and thrive at refrigeration temperatures (~4°C) suggests that their contribution to disease might be underappreciated. 

### 1.1. YE Infections

The major causative agent of yersiniosis is the gram-negative, zoonotic bacterial pathogen, YE, which is typically transmitted via the fecal-oral route [2]. The closely related YPT can also cause yersiniosis, but human YPT infections are less frequent than those caused by YE. Yersiniosis has been observed on all continents [3] but is most common in European countries. Some of the challenges associated with linking yersiniosis to its source of contamination are attributable to the heterogeneity of yersiniae populations within a plethora of environments and reservoirs including: soil, water, and a variety of animals. Yersiniosis is an important infection in European brown hares [4] and has additionally been detected in Canadian beavers, snowshoe hares, and muskrats [5]. Additionally, YE and YPT have been isolated form bats in Germany [6]. More relevant to humans is the prevalence of the yersiniae in animal food sources, particularly pigs and pork products [7–9], and more recently in domestic farm dogs in China [10]. Further complicating the picture of disease transmission, a recent study found that wild rodents on a European pig farm tested positive for YE, suggesting that rodents might serve as interspecies carriers between reservoirs [11]. YE has also been isolated from flies found in farm piggeries and kitchens [12], suggesting that arthropod vectors/insects might play a role in the transmission of the enteric yersiniae between animals and humans. Flies might also facilitate the spread of nosocomial infections which is of particular concern because there is at least one report of flies in Libyan hospitals carrying antibiotic-resistant strains of bacteria belonging to the *Enterobacteriaceae* family [13]. The major source of yersiniosis is swine, but recent isolates from contaminated chicken, milk, tofu, and water have also been reported [8, 14]. 

In healthy, immunocompetent individuals, yersiniosis symptoms range from mild, self-limiting diarrhea to mesenteric lymphadenitis. However, in immunocompromised individuals chronic conditions such as reactive arthritis have also been observed [15]. YE infection is generally established via digestion of contaminated food or water followed by bacterial adherence to small intestinal epithelial cells and eventual crossing of the intestinal barrier *via* M cells [16]. Subsequently, YE bacilli replicate in Peyer's patches and can sometimes spread to more distant lymphoid tissues, such as the mesenteric lymph nodes [16–18]. Dissemination from the distal ileum to the spleen and liver is relatively common, followed by extracellular replication and formation of monoclonal microabscesses [19]. The most common infection is acute gastroenteritis, mainly observed in children and infants on account of being somewhat immunocompromised due to an immature immune system. However, a host of other infections and complications can also occur in older children and adults, including pseudoappendicular syndrome, mycotic aneurysms [20–28], and, more rarely, sepsis as a secondary complication of yersiniosis or from blood transfusions. Several chronic conditions have also been described including: reactive arthritis, erythema nodosum, uveitis, glomerulonephritis, and myocarditis [3, 29]. While enteropathogenic yersiniosis is typically self-limiting in healthy individuals, the mortality rate can reach as high as 50% in immunocompromised persons, as a result of systemic bacterial dissemination [30].

### 1.2. YPT Infections

YPT causes zoonotic infections in a variety of hosts, including both wild and domestic animals and birds [31]. Human YPT infections, though less common than those caused by YE, are most often acquired from contaminated food or water [32]. Clinically, YPT infections typically present as abscess-forming mesenteric lymphadenitis and diarrhea but can also lead to secondary complications, such as perforation [33], subacute obstruction syndrome [34], intussusceptions [35], and acute renal failure [36] in rare cases. Additionally, patients with severe gastrointestinal bleeding in cases of YPT colitis have also been reported [37–39]. Similar to YE, the most common features of YPT infections in humans are ileocolitis and mesenteric lymphadenitis [40], the latter of which can affect appendix tissue and be mistaken for appendicitis [41]. YPT infections can be acute or chronic [42], with reticulogranulocytic infiltration, enlarged follicles, and necrosis with abscess formation in mesenteric lymph nodes [39, 43, 44]. Infection is usually self-limiting, but rare cases of sepsis can lead to a very high mortality rate (>75%) [45]. In addition to appendicitis, YPT infections have been confused with tumoral lesions [46], terminal ileitis, and Crohn's disease [47]. YPT has also been implicated in reactive arthritis, erythema nodosum, and Kwasaki autoimmune syndrome [48].

### 1.3. YE Epidemiology

Surveillance of human YPT infections is not routinely performed, and there are thus no complete databases from which information can be used to gauge trends in human YPT infections. However, there are several national surveillance networks that include yersiniosis in weekly, monthly, and yearly reports of human enteric disease cases/isolations, particularly those collected by member states of the European Union, the United States, and New Zealand. Potential sources of epidemiological data include clinical reports, laboratory isolations, sentinel site studies, reported cases, and rates calculated as cases per 100,000 persons in the affected population surveillance area per annum. Differences in reporting methods, isolation methods, and availability of strain information greatly complicate comparisons among countries and sometimes even among different regions/states/territories within an individual country. Furthermore, yersiniosis is infrequently monitored in developing countries, where enteric diseases are a major cause of infant and child mortality. For instance, the World Health Organization initiated a plan to address this issue in Africa in 1998 by working with member states and technical partners to implement the integrated disease surveillance and response (IDSR) program, but yersiniosis is not included as a primary surveillance target. Similarly, the Medical Sciences Center for Disease Control (http://www.moh.gov.cn), a division of China's Ministry of Health, reports communicable disease incidences on a weekly basis, but the plague is the only yersiniae-associated disease included in their surveillance efforts. 

**Figure 1:**
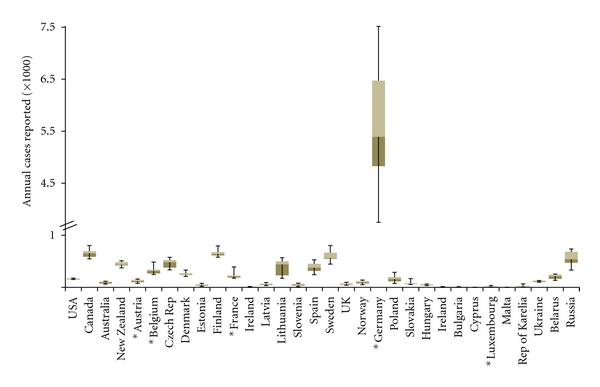
Human yersiniosis cases reported for selected countries that conduct active annual surveillance for the yersiniae. Surveillance data for years 2000 to 2009 were collected from national repositories for Canada (National Microbiology Laboratory, http://www.publichealth.gc.ca), the United States (FoodNet, http://www.cdc.gov/foodnet), 24 European Union members (European Food Safety Authority, http://www.efsa.europa.eu), New Zealand (The Institute of Environmental Science and Research, http://www.surv.esr.cri.nz), Australia (OZFoodNet, http://www.ozfoodnet.gov.au), Northwestern Russia, the Republic of Karelia, Ukraine, and Belarus (EpiNorth Project, http://www.ozfoodnet.gov.au). Russian data was obtained only from the following participating regions: Arkhangelsk oblast, Kaliningrad oblast, Leningrad oblast, Murmansk oblast, Nenets Autonomous okrug, Novgorod oblast, Pskov oblast, St. Petersburg City, Vologda oblast, and the Republic of Komi. For comparison, countries defined as Western European nations based on the classification scheme used by the United Nations include Austria, Belgium, France, Germany, and Luxembourg (which are marked with an asterisk). As shown, Germany reported the greatest number of human cases per annum for the ten-year period included (years 2000–2009), compared to all other countries examined, including the bordering countries of Denmark, Poland, Czech Republic, Austria, and France. The annual cases reported are shown on the *ordinate*, with the axis broken between 1,000 and 4,500 cases to allow the inclusion of Germany and other countries in one graphical display. Yearly cases were not adjusted for population differences. Individual countries are listed on the *abscissa*. USA: United States; UK: United Kingdom.

**Figure 2:**
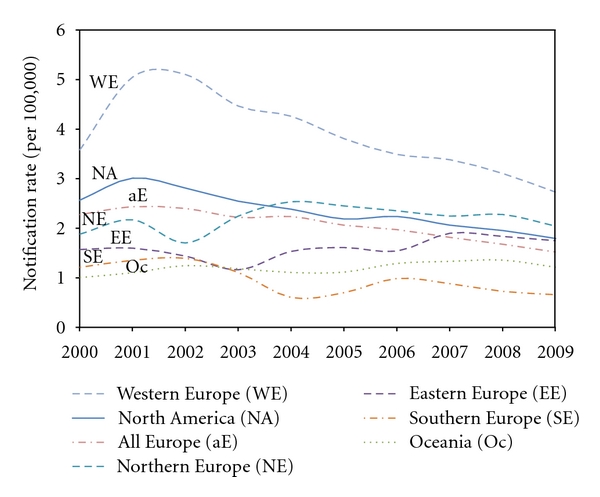
Line graph comparing the yearly incidence rate of yersiniosis reported for various European countries, North America, and Oceania. Surveillance data were collected from national repositories for Canada (National Microbiology Laboratory, http://www.publichealth.gc.ca), the United States (FoodNet, http://www.cdc.gov/foodnet), 24 European Union members (European Food Safety Authority, http://www.efsa.europa.eu), New Zealand (The Institute of Environmental Science and Research, http://www.surv.esr.cri.nz), Australia (OZFoodNet, http://www.ozfoodnet.gov.au), Northwestern Russia, the Republic of Karelia, Ukraine, and Belarus (EpiNorth Project, http://www.epinorth.org). The yearly incidence rate (cases per 100,000 in the surveillance population) was calculated based on total reported cases per year and published population figures included in published surveillance reports or governmental census sites. For countries where surveillance did not include the entire population, rates were adjusted based on the surveillance population and case information provided with the original surveillance data. For countries that did not provide data for all years included in the analysis (i.e., 2000–2009), the rate was extrapolated using linear regression (e.g., Canada, Australia, and Luxembourg). Notification rate (calculated as explained above) per 100,000 persons is shown on the *ordinate*, and a total of 30 countries presented by region are displayed on the *abscissa*. Western Europe (WE) includes Austria, Belgium, France, Germany, and Luxembourg. North America includes Canada and the United States. Northern Europe includes Latvia, Lithuania, Estonia, the United Kingdom, Ireland, Denmark, Norway, Finland, Sweden, and the Republic of Karelia. Eastern Europe includes the Czech Republic, Poland, Slovakia, Hungary, Bulgaria, the Ukraine, Belarus, and Northwestern Russia. Southern Europe includes Slovenia, Spain, and Malta. Oceania includes New Zealand and Australia. All of the available European data considered together (aE), representing a total of 28 countries, is also shown for comparison.

Despite the lack of surveillance in many countries, including Africa, Asia, the Middle East, Pacific Islands, Latin America, the Caribbean, and others, there are several national agencies in North America and Europe that provide yearly reports which include sporadic yersiniosis cases, outbreaks, and incidence rates in both humans and animals. As shown in Figure 1, there was a broad range of case reports for North America (including the US and Canada), Oceania (including Australia and New Zealand), and several European countries. For instance, Ireland reported between 3 and 14 isolations of YE/YPT from humans between the years of 2000 and 2009, while Germany reported between 3,906 and 7,186 confirmed cases of human yersiniosis during this same time period (Figure 1). Although, incidences have declined over the last 10 years (Figure 2), German yersiniosis cases account for more than half of all reported European yersiniosis events and ~90% of those within Western European nations that regularly surveyed their populations for YE-associated infections during the aforementioned ten-year-time frame (Figure 1). The reasons for the dramatically higher yersiniosis incidence rate in Germany compared to all other countries with active YE/YPT surveillance programs is unclear, but potential factors include variability in yersiniae isolation procedures and reporting systems, differences in clinical diagnostic frequency, degree of underreporting, prevalence of YE and YPT in animal reservoirs, differences in food processing, and variability in the consumption of meat products. There is some evidence to support the idea that higher meat consumption, particularly pork in Germany compared to other European nations might correlate with Germany's higher incidence of yersiniosis [49]. 

### 1.4. YE Genomics

YE is a heterogeneous group of organisms characterized by six biotypes and 60 serotypes. Biotypes can be distinguished based on level of pathogenicity, only one of which is nonpathogenic (Biotype 1A). “Old World” YE includes Biotypes 2–5, which are weakly pathogenic. Most virulent is the “New World” Biotype 1B, which is highly pathogenic to humans and lethal in a mouse model of infection [50]. Of the sixty serotypes of YE, only eleven have been associated with disease in humans, and the majority can be traced to only three commonly virulent serotypes: O:3, O:8, and O:9. These three serotypes are generally considered the causative agents of yersiniosis and vary based on geography. For instance, strain 1B/O:8 has been the predominant version of pathogenic YE in the United States [15]; in contrast, strain 3/O:9 is the most common cause of yersiniosis in China and in Europe [51, 52]. 

Isolates from these two pathogenic strains were sequenced [53, 54] and recently compared to identify common and unique virulence regions [54]. The results of this analysis indicated that the two strains share considerable genetic conservation/similarity, including most of the known YE virulence determinants. However, several 1B/O:8 key virulence regions were absent in the 3/O:9 strain [54] including high pathogenicity island (HPI) [55], *Yersinia* type II secretion 1 (*yts1*) [56], and the *Yersinia* Type III secretion apparatus (*ysa*). Likewise, the 3/O:9 strain possessed pathogenicity regions absent in the highly pathogenic 1B/O:8 strain. Strain 3/O:9-specific regions included a novel chromosomally encoded Type III secretion system (T3SS), ATP binding cassette transporter system, toxin-related gene clusters, and a flagellar gene cluster [54]. Sequencing additional YE strains, such as 4/O:3 that has recently emerged as an important cause of yersiniosis in the United States [57], will likely contribute to a better understanding of the relationship between strain-specific virulence factors and variations in clinical sequelae.

### 1.5. YPT Genomics

YPT can be classified into 14 distinct biotypes [58], five of which are almost exclusively pathogenic (O1–O5). The remaining nine biotypes (O6–O14) have been isolated from animals and the environment but never from human clinical samples [58–61]. Both pathogenic and nonpathogenic YPT can be further subdivided into 21 serotypes [62] based on the distribution of about 30 different O factors (O-specific polysaccharide of lipopolysaccharide [LPS]) within the species [58]. These serotypes vary geographically and in degree of pathogenicity [63], generally correlating with the size and presence of the chromosomal pathogenicity island, HPI [63]. Only Biotype O1 strains contain a complete, intact HPI. Biotype O3 contains a truncated version, and the pathogenicity island is entirely absent from all other YPT strains that have thus far been examined [64–66]. The pathogenicity of YPT depends on the presence of the T3SS-encoding virulence plasmid pYV [67], YPMa [68], and HPI [69] (described in detail in the next section), and clinical features are closely correlated with the various combinations of these three virulence factors. For instance, pYV is absent in one-fourth of the known virulent serotypes, which instead express the YPMa superantigen variant and/or HPI proteins [63]. The heterogeneous distribution of these factors accounts for the differences in clinical manifestations of infections in the Far East, Europe, and Western countries [63, 66, 70–72].

### 1.6. YE and YPT Virulence Factors

The genomes of YE, YPT, and YP are 97% identical, but the three bacteria cause vastly different diseases in humans, despite having a shared tropism for lymph nodes [73–76]. Their distributions of shared and unique virulence factors play a critical role in the different routes of infection, types of infections, and severity of disease in humans. Both chromosomal and plasmid-derived virulence factors play a role in yersiniae pathogenesis and in the establishment and progression of yersiniosis. YE pathogenicity depends on the presence of the 70-kb plasmid associated with *Yersinia* virulence, pYV [67, 77–79]. The pYV plasmid differentiates pathogenic from non-pathogenic strains, because it is essential for virulence [79]. The highly pathogenic *Y. enterocolitica* biotype 1B also harbors the chromosomal high-pathogenicity island (HPI), as do almost all European strains of *Y. pseudotuberculosis* serotype O1 [69]. HPI encodes proteins that are involved in the biosynthesis, regulation, and transport of the siderophore yersiniabactin [80, 81] and has thus been referred to as an “iron capture island” [63, 69]. There are five main genes within this island (*psn*, *irp1*, *irp2*, *ybtP*, and *ybtQ*) that are involved in the yersiniabactin system [80, 82, 83]. This system is positively regulated by YtbA, which is, itself, negatively regulated by the iron-responsive regulator Fur [84]. The *psn* and *irp2* genes are important for the high-pathogenicity phenotype of YPT [69, 85]. 

Almost all Far Eastern strains of YPT additionally produce one of three variants of a chromosomally encoded novel superantigenic toxin YPM (YPT*-*derived mitogen) encoded by the *ypm *gene [86, 87]. The original YPM (renamed YPMa) is encoded by *ypmA* [88] and plays a more important role in systemic infections than in gastroenteritis [68]. The other two variants, YPMb and YPMc, are encoded by the *ypmB* and *ypmC* genes, respectively [88, 89]. 

The small conserved RNA chaperone protein, Hfq is required for full virulence of a variety of pathogenic bacteria, including both YE and YPT [90]. Hfq is required for expression of the heat-stable enterotoxin Yst in YE [91]. In YPT, Hfq plays a role in the regulation of motility, intracellular survival, and production of T3SS effectors [90]. 

The YPT chromosomally encoded PhoP/Q system [92] regulates survival and growth in macrophages [93, 94] and covalent modifications of LPS that reduce its stimulatory capacity [95], thereby empowering bacteria to avoid, minimize, or delay macrophage activation. In a mouse model of intestinal infection, mutants devoid of PhoP were 100-fold attenuated in virulence due to a reduced capacity to survive and replicate intracellularly within macrophages [93]. The global PhoPQ regulon also senses the reduction in Mg^2+^ and possibly Mn^2+^ levels that characterizes the intracellular environment of host cells. MntH, a putative *Yersinia* Mn^2+^ transporter, was recently proposed to promote survival of the bacteria within phagocytic vacuoles by protecting them from reactive oxygen species [96].

### 1.7. Establishment of Yersiniosis Infection

**Figure 3:**
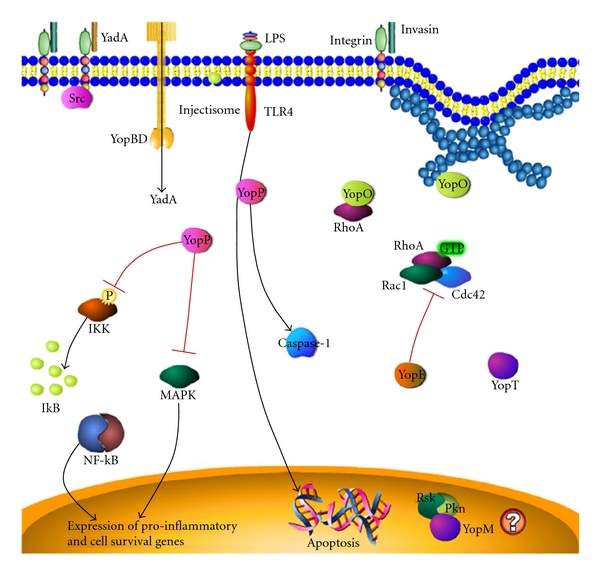
Mechanisms of action of the enteropathogenic yersiniae Ysc T3SS effectors (Yops) on host cell signaling and survival. As shown, membrane-bound *Yersinia *Yad and invasin proteins bind host cell *β*1-integrins, bringing the bacteria into close proximity to the host cell thereby facilitating insertion of the T3SS injectisome needle-like structure into the targeted host cell. Yops are then translocated across the host plasma membrane and into the cytoplasm, where they interact with the cytoskeleton and host cell signaling molecules. YopO/YpkA interacts directly with the cytoskeleton, as well as the small GTPase signaling molecules, RhoA, Rac1, and Cdc42. YopE inhibits the activities of RhoA, Rac1, and Cdc42. YopP/J promotes LPS-induced host cell apoptosis and directly induces capsase-1 cleavage. YopP/J also inhibits mitogen-activated protein kinases (MAPK) and IKK-mediated NF-*κ*B activation, which prevents expression of proinflammatory and cell survival genes. YopM forms a complex with Rsk and Pkn in the host cell nucleus, which is believed to contribute to bacterial pathogenesis. The figure was produced using Pathway Builder 1.0, a cell signaling drawing tool provided through the Protein Lounge (http://www.ProteinLounge.com).

In many pathogens, virulence factors are closely coupled to temperature, and this temperature regulation is particularly important for the establishment of infection. At environmental temperatures (less than 28°C) and under acidic conditions at 37°C, the enteric yersiniae optimally express the invasin protein, which is encoded by the chromosomal *inv locus* [17, 18]. Upon ingestion, invasin binds to B1 integrins on host cells and facilitates penetration of the epithelial layer (Figure 3). The gradual increase in temperature within the host induces the expression of virulence factors necessary to establish a stronghold within the lymph tissues and evade immune system detection. Expression of the chromosomal *ail* (*attachment invasion*)* locus*, for instance, is induced at 37°C, and the resulting Ail/OmpX protein further enhances epithelial cell invasion. Establishment of infection also requires translocation of toxic effectors via a T3SS as well as “other transporter systems” [97]. Regulation of adherence and invasion is mediated via the regulator of virulence A (RovA), which positively regulates *inv* expression, *Yersinia*-modulating protein (YmoA), and histone-like nucleoid structuring protein (H-NS) [98–103]. 

*Yersinia* adhesion A protein (YadA) also mediates mucus and epithelial cell attachment and, in concert with invasin, promotes host cell invasion (Figure 3). YadA is a multifunctional, surface-exposed virulence factor encoded on the pYV virulence plasmid that confers the ability to adhere to extracellular matrix proteins [104–106]. Induction of YadA expression is coordinated with the upregulation of Yops (*Yersinia* outer membrane proteins) [107, 108]. The contribution of YadA to virulence is greater for YE than for YPT, playing a significant role in the positive regulation of both adherence to and invasion of host cells [105, 109]. YadA plays only a minor role in YPT, conferring merely an adhesive phenotype [110–112]. Similar to invasin, YadA initiates internalization by binding to extracellular fibronectin that is bound to a 5b1 integrin [105]. YadA from YPT and YE binds fibronectin, collagen I, II, and IV, and laminin, albeit with different affinities thus promoting variable virulence properties [105]. YadA elicits an inflammatory response in epithelial cells by inducing mitogen-activated protein kinase-(MAPK-) dependent interleukin (IL)-8 production and by contributing to the resulting intestinal inflammatory cascade [113, 114]. Interaction of YadA with collagen has been proposed to contribute to chronic yersiniosis infections, such as the development of reactive arthritis [113–116] which has been demonstrated in a rat model [117–119]. 

In addition to inhibition and invasion of host cells, both Ail and YadA play significant roles in complement resistance and immune evasion. Ail and YadA inhibit the alternative complement pathway by binding regulator factor H and usurping its natural function to prevent lysis of host cells [120–123]. Ail and YadA similarly subvert the classical complement and lectin pathways by binding to C4b-binding protein, thereby promoting the degradation of the C4b complement factor and preventing the formation of the C3 convertase that would otherwise lead to lysis of the bacterial cells [123]. 

Other YPT virulence factors include the putative DNA adenine methyltransferase, YamA, which is required for full virulence [124], and several proteins that aid in bacterial survival under acidic conditions. An aspartate-dependent acid survival system was recently described for YPT, which plays a role in bacterial survival and thus facilitates establishment of infection [125]. A drop in pH induces the expression of the YPT *aspertase* (*aspA*) gene; the encoded gene product, AspA, subsequently produces ammonia, allowing the ingested organisms to survive the acidic gastrointestinal environment [125]. Other bacterial factors that promote survival under acidic conditions include urease [126], TatC [127], PhoP, OmpR, and PmrA [128, 129]. Acidic pH also induces a downregulation of the transcriptional regulator, Cra (for catabolite repressor/activator), which increases bacterial acid survival [130]. Presumably Cra mediates this action via transcriptional regulation, but its mechanism of action remains unknown.

### 1.8. T3SS and Yop Effectors

The T3SS, which is encoded on the pYV virulence plasmid and is common to all three pathogenic yersiniae, plays a substantial role in both the establishment and outcome of infection. The T3SS injectisome spans both the inner and outer bacterial membranes, and virulent effector proteins, termed *Yersinia* outer proteins (Yops), are translocated through a host-cell docked *Yersinia* secretion protein F (YscF) needle, directly into the targeted host cells [131]. The YopB and YopD proteins form a pore in the host cell plasma membrane, allowing for the docking of the YscF needle and eventual translocation of the effectors (Figure 3). Proper assembly of a stable injectisome complex also requires the YscE and YscG cytosolic chaperone proteins [132]. There are six effector Yop proteins (YopE, YopH, YopP/J, YopO/YpkA, and YopM) that mediate immune evasion by interfering with host signal transduction pathways, disruption of the host actin cytoskeleton, and by inducing host-cell apoptosis (Figure 3) [133, 134]. 

Delivery of Yops requires close contact between the bacterial and host cells and is mediated by YadA and invasin through their binding to *β*1-integrins (Figure 3) [135, 136], which when stimulated cause the activation of Src kinases and RhoA that facilitate Yop translocation via modulation of actin polymerization [137]. In the absence of Yops, activation of *β*1-integrins would instead lead to actin rearrangements that promote bacterial internalization [138]. Each Yop has a designated chaperone called a Syc protein (for specific Yop chaperone) (e.g., SycE for YopE), required for Yop secretion [133]. The T3SS injectisome is triggered by host-cell contact [139], as well as *in vitro* by temperature (37°C) and low calcium conditions (which serve to emulate intracellular conditions of the host cells) [140–142]. Yop effectors allow evasion of immune responses by blocking host phagocytic function [133, 143, 144], which is vital for bacterial replication and intracellular survival. The *Yersinia* T3SS pore itself was recently suggested to trigger processing of IL-1*β* and IL-18 in macrophages [75, 145] and subsequent formation of an inflammasome, a cytosolic innate immune complex [146] that triggers inflammation and pyroptosis in response to pore formation [147, 148].

Host cell death is mediated by the YopP/J effector, a serine-threonine acetyltransferase that induces apoptosis of phagocytes by modulating the actions of LPS (Figure 3). Upon binding to the toll-like receptor (TLR)-4, LPS induces the activation of proapoptotic host factors via TRIL (Toll/IL-1 receptor domain-containing adapter inducing IFN-*β*) [149, 150], while simultaneously downregulating proinflammatory and cell survival genes via inactivation of MAPK and nuclear factor kappa B (NF-*κ*B) transcription factor (Figure 3) [151–153]. YopP/J specifically inhibits the inflammatory and cell survival actions of LPS [154, 155], thus tipping the scale towards host cell apoptosis [150, 156]. YopP/J-mediated inhibition of host cell proinflammatory responses involves inhibition of IKK*β* activation, and thus NF-*κ*B activity (Figure 3) [157], which results in the reduction of TNF-*α* release by macrophages [158], prevention of IL-8 secretion by epithelial cells [155], and reduction in the presentation of ICAM-1 and E-selectin adhesion factors on the surface of epithelial cells [159]. More recently, it was shown that YopP/J also directly activates caspases (Figure 3) independently of upstream death receptors [160–162]. 

Once injected into the host-cell cytoplasm, YopE, -H, -P, and -T cooperatively disrupt the cytoskeleton of epithelial cells, macrophages, and dendritic cells thereby decreasing their capacity to engulf the invading bacteria. YopP/J can also facilitate evasion of adaptive immune responses by inhibiting the ability of dendritic cells to present antigens to CD8^+^ T cells [163], either directly or possibly by decreasing the population of dendritic cells via induction of apoptosis [162, 164, 165]. A similar strategy is employed by YPT using the GTPase activating protein (GAP), YopE, to circumvent phagocytosis by dendritic cells [163, 166]. In addition to the *Yersinia* injectisome and effector proteins, at least three adaptor proteins YopB, YopD, and VirF/LcrV (low calcium response V antigen) are required for T3SS activity [133]. VirF/LcrV (also called V antigen) is a multiple adaptational response (MAR) family member that regulates the T3SS at the level of transcription and, when secreted into the extracellular host environment, contributes to virulence by downregulating inflammation [167, 168].

YopE, YopT, and YopO/YpkA counteract host-cell phagocytosis by acting on monomeric Rho GTPases responsible for regulation of cytoskeleton dynamics [133]. YopE exhibits GAP activity, thereby inducing GTP hydrolysis and, thus, inactivation of RhoA, Rac1, and Cdc42 (Figure 3) [169–171]. YopT, on the other hand, acts as a cysteine protease that inactivates Rho, Rac, and Cdc42 *via* cleavage [172, 173]. YopO/YpkA is a serine-threonine kinase with sequence and structural similarity to RhoA-binding kinases that undergoes autophosphorylation upon binding to actin [174–176]. YopO can also bind directly to RhoA and Rac-1 with currently unknown consequences [133]. 

The YopH effector was also recently shown to inhibit host inflammatory responses *via *the downregulation of chemokine monocyte chemoattractant protein 1 (MCP-1) [177]. YopH of YPT inhibits activation of the phosphatidylinositol 3-kinase pathway, resulting in the prevention of antigen-mediated activation of lymphocytes [177, 178]. YopH, a protein tyrosine phosphatase, disrupts T-cell and B-cell activation by interfering with phosphorylation signaling events resulting in decreased expression of the costimulatory molecules B7.2 and CD69, as well as the leukocyte mitogen, IL-2 [178, 179]. Very little is known about YopM, but its deletion results in a dramatic decrease in virulence [180]. YopM appears to be injected into host cells, along with other T3SS effector proteins [181], but there is also evidence that YopM can bind to the extracellular acute phase protein *α*1-antitrypsin [182]. More recently, YopM was shown to form a complex with ribosomal S6 kinase (RSK) and protease-activated kinase (PKN) (Figure 3) [183], which results in sustained activation of RSK and possibly contributes to *Yersinia* pathogenicity [184, 185].

### 1.9. Chromosomal T3SSs

In addition to the pYV-encoded T3SS, there are two additional chromosomally encoded T3SSs in YE: a flagellar T3SS and the Ysa T3SS [186, 187]. The Ysa T3SS is optimally expressed under high salt concentrations, 26°C, and at stationary growth phase [186, 188, 189]. Salt responsiveness is mediated by the sycByspBCDA operon, which is regulated by YsaE and the SycB chaperone [189]. The Ysa T3SS plays a role in virulence [186] and is important for colonization of the small intestine despite its optimal expression at non mammalian temperatures (26°C) [190]. There are 15 known Ysa effector proteins (Ysps), which are thought to function similarly to Yop effectors as modulators of host immune responses [191]. Interestingly, the flagellar T3SS, which functions in the biogenesis of flagella, secretes Fop effectors that also play a role in the pathogenesis of YE [187]. YplA (*Yersinia* phospholipase A), for instance, is a Fop required for colonization of Peyer's Patches and mesenteric lymph nodes that contributes to inflammatory responses within these tissues [192].

### 1.10. Type VI and IV Secretion System

T3SSs are not the sole secretion systems identified in the yersiniae that promote bacterial virulence. In fact, a type VI secretion system (T6SS) was recently identified in YPT, which harbors four copies, one of which was recently shown to be regulated by temperature, growth phase, and the N-acyl homeserine lactone-AHL-dependent quorum sensing system [193]. YPT also harbors a type IV pilus gene cluster that contributes to pathogenicity [194].

### 1.11. Host Responses to YE and YPT Infection

*Yersinia* infections are biphasic and are initiated by a “quiet” 36–48 hour period of bacterial replication without a measurable host response. This initial “quiet” phase is followed by an influx of activated phagocytes into infected tissues and lymph nodes, which induces an acute inflammatory response characterized by cytokine production and tissue necrosis [74, 76, 195–199]. The T3SS Yop effectors are likely responsible for the initial inhibition of phagocytic functions, but the mechanisms behind such a sudden, bipolar “off-on” inflammatory response are presently not fully understood. The T3SS is absolutely required for effective colonization of systemic organs, and T3SS inactivation leads to rapid clearance of the bacteria by the host [200–202]. As a result, yersiniae lacking a functional T3SS are avirulent and can function as live attenuated vaccine strains in mice [200, 203, 204].

Recent evidence suggests that macrophages can compensate for YopE/YopH-mediated inhibition of the endosomal MHC class II antigen presentation pathway by an autophagy-dependent mechanism [205]. Thus, autophagy might serve as an alternative counter-pathway by which the host might mount an MHC class II-restricted CD4^+^ T-cell response against *Yersinia* T3SS-mediated translocation of Yop virulence effectors [205]. However, whereas Deuretzbacher et al. [206] demonstrated autophagy-mediated degradation of macrophage internalized YE, YPT was shown to usurp the autophagosome pathway for continued replication within macrophages at the intestinal site of infection [207]. 

Murine studies have demonstrated that CD4^+^ and CD8^+^ T cells are required for control of YE infection [196, 208], as are IFN-*γ*-mediated Th1 immune responses, including macrophage production of TNF, IL-12, and IL-18 [209–212]. Inhibition of T-cell proliferation and dendritic cell functions by Yops are primary mechanisms by which the yersiniae evade both innate and adaptive immune responses [213]. Interestingly, the yersiniae induce both apoptosis of naïve macrophages and inflammatory cell death (pyroptosis) of activated macrophages, which is consistent with its biphasic infection process [73, 75]. Increased inflammation associated with the redirected host cell death could initially benefit the yersiniae but later could contribute to a generalized immune response and eventual clearance of bacteria [73, 75].

### 1.12. Detection and Prevention of Food-Borne Yersiniosis

YE and YPT clinical infections most often occur following ingestion of the bacteria in contaminated food or water. The two aforementioned yersiniae have been isolated from meat, fresh produce, and milk, but their presence is frequently unapparent due to detection difficulties. Various YE strains are most often distinguished by pulsed-field gel electrophoresis (PFGE), but there is currently no standardized test or database for consistent identification. Moreover, enteropathogenic *Yersinia* species are not included in the protocols that are used by laboratories in PulseNet which, in cooperation with the Association of Public Health Laboratories (APHL), coordinates with public health laboratories to subtype bacterial foodborne pathogens [214]. The heterogeneity of both YE and YPT makes definitive detection difficult, and PFGE produces multiple bands that are not especially distinctive based on serotype [29, 215–217]. Some reports have suggested that current detection methods can produce false-negatives or false-positives based on variability in the presence of *Yersinia* virulence factors, and their variable correlation with pathogenicity [218, 219]. Suggestions for improving detection include the use of more than one restriction nuclease in PFGE analyses [29] and application of a recently developed multilocus variable-number tandem-repeat analysis (MLVA) for YE [220, 221].

Detection is an especially important concern, because both YE and YPT can readily proliferate at refrigeration temperatures (4°C) and even as low as 0°C. Furthermore, the enteropathogenic yersiniae can likewise adapt to and thrive under modified atmospheric conditions that are often used in conjunction with colder temperatures as common methods of food preservation. Survival and cell growth at low temperatures are accomplished via a short-term, cold-shock response, in which a variety of stress response proteins are produced that mediate bacterial adaptation to the sudden drop in temperature (reviewed in [222]). Both YE and YPT are also capable of more long-term cold adaptation, a process that requires polynucleotide phosphorylase (PNPase), a cold-shock exoribonuclease that enhances both T3SS function as well as promoting growth under cold conditions [223]. 

Pathogenic YE produce insecticidal toxins, encoded by *tc* (*toxin complex-like*) genes located within a chromosomal pathogenicity island [224, 225]. These insecticidal toxins are expressed at low temperatures [226], but they are nonetheless thought to possess virulence functions in mammalian hosts [224, 225]. It is possible that the presence of these insecticide toxins suggests that the normal life cycle of YE includes an insect stage, as previously proposed [226], and these toxins might facilitate growth of the organisms in refrigerated food products. Tc proteins in YPT, on the other hand, do not possess insecticide activity but rather confer toxicity to mammalian cells [227] and might, therefore, play a role in human disease. 

The presence of *β*-lactamases that confer antibiotic resistance to some pathogenic strains of YE [228, 229] underscores the importance of surveillance for these pathogenic organisms. While these organisms are not monitored nationally, yersiniosis incidence rates and patient demographics in the United States are collected annually by the Foodborne Diseases Active Surveillance Network (FoodNet). FoodNet reported 1,355 and 18 human yersiniosis cases of YE and YPT, respectively, in the U.S. between 1996 and 2007. However, based on FoodNet's assessments [230], cases of yersiniosis, especially those caused by YPT, are likely under-estimated in the U.S. due to lack of testing and difficulty associated with culturing the yersiniae on standard media [231, 232].

## 2. Conclusions

YE is the major cause of yersiniosis in humans, although prevalence of YPT-associated disease is likely underreported due to lack of surveillance and differences in applied isolation strategies. Extreme heterogeneity among strains of YE and YPT further complicates efforts to link contamination to the source and monitor human disease in a uniform manner comparable to other more thoroughly studied food-borne pathogens (e.g., *Salmonella*). Although a plethora of animal hosts serve as reservoirs for both YE and YPT, human disease-associated yersiniae are most prevalent in swine. In healthy individuals, the resulting illness can manifest as mild, self-limiting diarrhea, but in young children and immunocompromised individuals yersiniosis can represent a significant source of morbidity and mortality. Additionally, chronic diseases, such as reactive arthritis and secondary (or nosocomially derived) complications such as sepsis, can develop in immune compromised persons.

YE and YPT are heterogeneous organisms that differ in genomic content and degree of pathogenicity. Two pathogenic strains (1B/O:8 and 3/O:9) have been sequenced and compared [53, 54] to gain insight into virulence mechanisms required to initiate infection and cause acute symptoms or chronic conditions in patients. YE infection is generally established via consumption of contaminated food or water and involves adherence to and translocation across the intestinal barrier via M cells [16]. Other virulence factors include the pYV plasmid, which encodes a T3SS essential for YE pathogenicity [79], and the chromosomal HPI locus found in highly pathogenic strains [69]. Pathogenic YPT strains encode a novel superantigenic toxin, YPM that contributes to systemic infections [68] and a PhoP/Q system important for regulation of bacterial survival and growth within macrophages [93, 94]. Type IV pilus genes [194] and a recently discovered T6SS [193] also contribute to yersiniae virulence. While a great deal of molecular work has contributed significantly to a better understanding of YE and YPT pathogenicity, there is much to be gained from future studies, particularly those aimed at dissecting the contributions of various virulence factor combinations to pathogenicity, the resulting type of infection, and ability of the host immune system to clear the bacteria. Very little is known about yersiniae-associated autoimmune disease and other chronic conditions. For instance, YPT is much less studied than YE and thus might be underappreciated as a causative agent of yersiniosis. As such, yersiniosis surveillance efforts concentrate almost exclusively on YE, making attempts to accurately estimate YPT-associated gastroenteritis incidence nearly impossible. 

Enteropathogenic YE and YPT cause yersiniosis globally and are of significant concern to the pork industry. The ability of the enteropathogenic yersiniae to replicate and thrive at refrigeration temperatures, coupled with their seemingly ubiquitous nature, suggests that future and more uniform surveillance measures are inevitable and requisite. At present, enteropathogenic yersiniae cases are likely underestimated; however, recent preventative measures in the pork industry and increased attention, both in the research laboratories and clinics, will provide much needed insight and better strategies for managing yersiniosis. Furthermore, more thorough and uniform surveillance measures will allow us to more accurately gauge national and global yersiniosis trends and better predict which agricultural, hygienic, and clinical efforts are effective in reducing the incidence of yersiniosis infection in the general population.
